# Worsening Mitral Stenosis Due to Post-transcatheter Aortic Valve Replacement Improvement of Concomitant Aortic Insufficiency

**DOI:** 10.7759/cureus.4424

**Published:** 2019-04-10

**Authors:** Hiren Patel, Muhammad Hamza Saad Shaukat, Mikhail Torosoff, Steven Fein, Anthony Nappi

**Affiliations:** 1 Cardiology, Albany Medical Center Hospital, Albany, USA; 2 Internal Medicine, Albany Medical Center Hospital, Albany, USA

**Keywords:** tavr, aortic regurgitation, mitral stenosis

## Abstract

Transcatheter aortic valve replacement (TAVR) has become an alternative to surgical treatment in severe aortic valve stenosis, with expanding indications and rapidly growing number of performed procedures. Poor opposition of TAVR prosthesis to the aortic root causes paravalvular leak, while mitral regurgitation and stenosis have been associated with valve implantation low in the left ventricular outflow tract (LVOT). We report an unusual case of a patient with combined severe aortic stenosis and moderate aortic insufficiency who underwent elective TAVR, which resulted in significant increase of a pre-existing mitral valve gradient. Rapid post-TAVR increase in mitral valve gradient was likely due to improvement in aortic regurgitation and decreased left ventricular end-diastolic pressure (LVEDP).

## Introduction

Transcatheter aortic valve replacement (TAVR) has become an alternative to surgical treatment in severe aortic valve stenosis, with expanding indications and rapidly growing number of performed procedures. Poor opposition of TAVR prosthesis to the aortic root causes paravalvular leak, while mitral regurgitation and stenosis have been associated with valve implantation low in the left ventricular outflow tract (LVOT) [1-2]. We report an unusual case of a patient with combined severe aortic stenosis and moderate insufficiency who underwent elective TAVR, which resulted in significant increase of a pre-existing mitral valve gradient. Rapid post-TAVR increase in mitral valve gradient was likely due to improvement in aortic regurgitation and decreased left ventricular end-diastolic pressure (LVEDP).

## Case presentation

**Figure 1:**
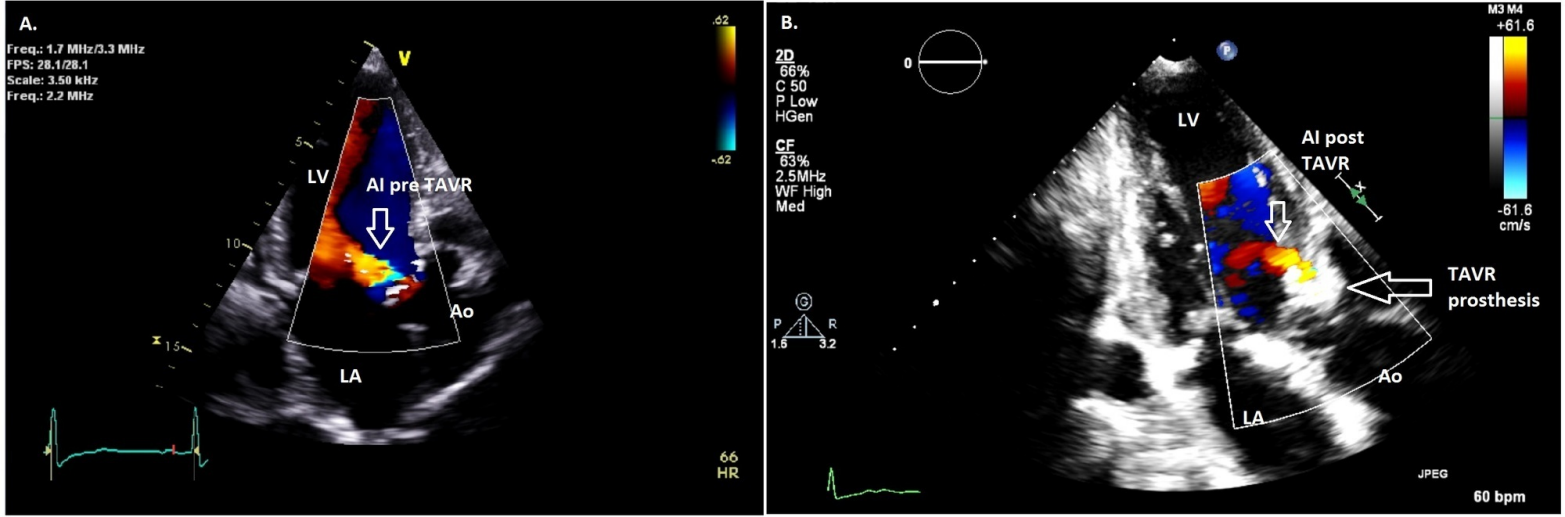
Improvement in severity of aortic insufficiency on Doppler echocardiography - before (A) and after transcatheter aortic valve replacement (TAVR) (B).

A 77-year-old male with symptomatic severe calcific aortic valve stenosis was referred for an elective TAVR. ECHO at presentation, in addition to severe aortic stenosis, showed moderate aortic insufficiency (Figure 1A), thickened and calcified mitral valve with mild transvalvular gradient (3.5 mmHg). The patient underwent a transfemoral TAVR with a 29-mm Sapien 3 valve (Edwards Lifesciences, Irvine, CA). The 24-h follow-up transthoracic echocardiogram performed on the patient in stable clinical condition, with the heart rate of 60 bpm, revealed well-seated aortic valve prosthesis with a significant improvement in aortic insufficiency (Figure 1B). However, despite stable hemodynamics and normal heart rate, there was a significant worsening in the mitral valve stenosis parameters: mean trans-mitral pressure gradient increased from 3.5 to 9 mmHg, mitral valve peak velocity increased from 149 to 238 cm/s, and pressure half-time increased from 110 to 170 ms.

It is well known that mitral stenosis severity/gradient may be underestimated in patients with elevated LVEDP secondary to significant aortic insufficiency. We postulate that successful treatment of aortic valve stenosis and insufficiency with TAVR led to a decline in LVEDP which unmasked pre-existing significant mitral valve stenosis.

## Discussion

Increased pressure gradient across the mitral valve is uncommon in post-TAVR patients without significant pre-existing mitral stenosis. When noted, increased mitral valve gradient is typically attributed to post-procedure anemia and tachycardia, which increase flow across the stenotic mitral valve and decrease duration of diastole [3]. In some patients post-TAVR mitral stenosis is iatrogenic, with restricted diastolic opening of the anterior mitral valve leaflet caused by impingement by the low-placed aortic valve prosthesis [4-6]. When severe and associated with acute heart failure, iatrogenic post-TAVR mitral stenosis may necessitate an urgent extraction of the aortic valve prosthesis and surgical aortic valve replacement [5]. In initially stable patients, there may be a progressive worsening of iatrogenic mitral stenosis leading to heart failure symptoms within 12 months post-TAVR [6].

Here we present an unusual case of combined severe aortic valve stenosis, moderate aortic valve insufficiency, and fibrocalcific mitral valve disease with only mild pre-TAVR mitral valve gradient. Following a successful TAVR with improvement in aortic stenosis and insufficiency, the patient experienced a significant increase in the mitral valve gradient. However, the aortic valve prosthesis did not impinge on the mitral valve, the heart rate was not elevated, and patient did not suffer a significant blood loss. We conclude that in our patient, successful treatment of aortic valve stenosis and insufficiency with TAVR led to significant decline in LVEDP with associated increase in mitral valve gradient, revealing true extent of the pre-existing significant mitral valve stenosis. This complication is not preventable. However, identification of post-TAVR mitral stenosis is important because of the need for close long-term follow up with repeat echocardiography to monitor changes in the mitral gradient. Long-term mitral disease may progress and, with improvement in LV hypertrophy expected after aortic valve replacement [7], there may be further decline in the LVEDP and associated increase in mitral valve gradient.

## Conclusions

In post-TAVR patients, mitral valve stenosis is typically ascribed to restricted anterior mitral leaflet motion caused by a low-placed AV prosthesis. However, as our case illustrates, in patients with pre-TAVR combined aortic valve stenosis and insufficiency, post-AVR amelioration of aortic valve regurgitation may be associated with increased mitral valve gradient commensurate with true degree of mitral stenosis.
